# Soot Monitoring of Gasoline Particulate Filters Using a Radio-Frequency-Based Sensor

**DOI:** 10.3390/s23187861

**Published:** 2023-09-13

**Authors:** Stefanie Walter, Peter Schwanzer, Gunter Hagen, Hans-Peter Rabl, Markus Dietrich, Ralf Moos

**Affiliations:** 1 Bayreuth Engine Research Center (BERC), Department of Functional Materials, University of Bayreuth, 95447 Bayreuth, Germany; 2 Combustion Engines and Emissions Control Laboratory, Ostbayerische Technische Hochschule Regensburg, 93053 Regensburg, Germany; 3 Vitesco Technologies GmbH, 93055 Regensburg, Germany

**Keywords:** gasoline particulate filter (GPF), radio-frequency (RF), soot mass determination, engine test bench, microwave cavity perturbation, dielectric properties

## Abstract

Owing to increasingly stringent emission limits, particulate filters have become mandatory for gasoline-engine vehicles. Monitoring their soot loading is necessary for error-free operation. The state-of-the-art differential pressure sensors suffer from inaccuracies due to small amounts of stored soot combined with exhaust gas conditions that lead to partial regeneration. As an alternative approach, radio-frequency-based (RF) sensors can accurately measure the soot loading, even under these conditions, by detecting soot through its dielectric properties. However, they face a different challenge as their sensitivity may depend on the engine operation conditions during soot formation. In this article, this influence is evaluated in more detail. Various soot samples were generated on an engine test bench. Their dielectric properties were measured using the microwave cavity perturbation (MCP) method and compared with the corresponding sensitivity of the RF sensor determined on a lab test bench. Both showed similar behavior. The values for the soot samples themselves, however, differed significantly from each other. A way to correct for this cross-sensitivity was found in the influence of exhaust gas humidity on the RF sensor, which can be correlated with the engine load. By evaluating this influence during significant humidity changes, such as fuel cuts, it could be used to correct the influence of the engineon the RF sensor.

## 1. Introduction

In recent years, the transition from conventional port fuel injection (PFI) to gasoline direct injection (GDI) has significantly reduced fuel consumption and, thus, CO_2_ emissions in gasoline engines. However, GDI engines produce higher particulate emissions [1]. Particulate emissions are not only harmful for health reasons [2], but also because soot particles contribute significantly to global warming [3]. For this reason, emission limits will be further tightened in the coming years with the introduction of the Euro 7 emissions standards. Internal engine measures alone are already unable to reduce particulate emissions sufficiently to meet the current emission standards. For this reason, the majority of GDI vehicles in production today are equipped with gasoline particulate filters (GPFs) [4,5]. To ensure error-free operation of particulate filters, the stored particulate mass must be monitored, because it affects the filtration efficiency of the GPF, as well as the engine operation via the generated backpressure.

For this purpose, diesel engines are equipped with a differential pressure sensor according to the state-of-the-art. In gasoline engines, the differential pressure sensor is significantly less accurate due to altered operating conditions, such as lower amounts of stored soot, higher exhaust gas volume flow, higher exhaust temperatures, or partial regeneration, resulting in a changed soot distribution within the filter [6,7]. An alternative approach is the radio-frequency-based filter diagnosis (RF sensor), which detects the filter loading based on a change in the dielectric properties of the filter caused by soot deposition. Its functionality in gasoline applications has already been demonstrated [8]. However, it was found that the sensitivity of the RF sensor can depend on engine operation during soot formation [9].

The aim of this work is now to investigate the influence of engine operation on the dielectric properties of soot particles and whether this can be corrected. In addition, it investigates whether the resulting sensitivity of the RF sensor can be directly derived from the dielectric properties of the soot. For these investigations, GPFs were loaded on an engine test bench under various conditions. The microwave cavity perturbation (MCP) method was used to determine the dielectric properties of the soot particles. In order to analyze the influence of soot loading on the RF sensor under controlled conditions and to compare its sensitivity with the dielectric properties of soot, soot-loaded filters were then analyzed on a lab test bench, at which exhaust conditions can be simulated.

## 2. Fundamentals of the RF-Based Measurement Systems

Both radio-frequency-based measurement methods used in this work—microwave cavity perturbation (MCP) and the RF sensor—are based on the different propagation of electromagnetic waves depending on the properties of the permeated medium. The electromagnetic waves are excited within a cavity surrounded by conductive walls—also known as a resonator—where they can form standing waves by mutual superposition. The resulting resonant modes are then influenced by the properties of the materials in the cavity [10].

The dielectric properties of materials can be described by the complex relative permittivity $\varepsilon_{r}$, according to Equation (1) [10]. Its real part $\varepsilon_{r}^{\prime }$, often only referred to as permittivity, is a measure of the polarization fields occurring in the material. The imaginary part $\varepsilon_{r}^{\prime \prime }$ is composed of two different loss measures and describes the dielectric losses that lead to the attenuation of the electromagnetic wave. The losses $\varepsilon_{r,\mathrm{pol}}^{\prime \prime }$ are caused by dipoles, which oscillate due to the polarization fields. In addition to these, losses occur in electrically or ionically conductive materials, depending on the material conductivity *σ*, the angular frequency of the electromagnetic wave ω, and the vacuum permittivity $\varepsilon_{0}$.

(1) $$\varepsilon_{r}=\varepsilon_{r}^{\prime }-{j\varepsilon }_{r}^{\prime \prime }=\varepsilon_{r}^{\prime }-j\left(\varepsilon_{r,\mathrm{pol}}^{\prime \prime }+\frac{\sigma }{\varepsilon_{0\;}\omega }\right)$$

The electromagnetic wave is usually coupled into the resonator via coupling elements, such as antennas. The resulting resonances can be evaluated based on the scattering parameter *S*_21_ measured by a vector network analyzer (VNA) and characterized by two parameters: the resonant frequency $f_{\mathrm{res}}$ and the quality factor *Q*. These parameters are evaluated in this paper using the same methods as those used in [8,11].

### 2.1. Determination of Dielectric Properties Using the Microwave Cavity Perturbation

By knowing the resonant frequency and the quality factor, the dielectric properties of the material located in a resonator can be derived using the microwave cavity perturbation (MCP) method [12]. For this method, a small sample of material compared with the resonator volume is introduced into the cavity. This results in a shift in the resonant frequency ${\Delta f}_{\mathrm{rel}}$ caused by the sample permittivity $\varepsilon_{r}^{\prime }$ and a change in the inverse quality factor ${\Delta Q}^{-1}$ caused by the sample losses $\varepsilon_{r}^{\prime \prime }$ according to Equations (2) and (3). The magnitude of the resonant shift depends not only on the sample volume $V_{s}$, but also on the effective resonator volume $V_{\mathrm{eff}}$, which takes into account the field distribution of the excited resonant mode [12,13]. The resonant parameters before inserting the sample are denoted by $f_{0}$ and $Q_{0}$. The parameters with the sample inserted are denoted by $f_{s}$ and $Q_{s}$.

(2) $${\Delta f}_{\mathrm{rel}}=\frac{f_{0}-f_{s}}{f_{0}}=\left(\varepsilon_{r}^{\prime }-1\right)\;\frac{V_{s}}{{2V}_{\mathrm{eff}}}$$

(3) $${\Delta Q}^{-1}=\frac{1}{Q_{s}}-\frac{1}{Q_{0}}=\varepsilon_{r}^{\prime \prime }\;\frac{V_{s}}{V_{\mathrm{eff}}}$$

However, the described correlations are only valid under the condition that the electromagnetic field in the resonator is almost not disturbed due to the insertion of the sample. This is not the case for comparatively large sample volumes or high dielectric losses. However, it is possible to correct for these perturbations [14,15]. The corrections made in [14] concerning depolarization behavior and field distribution allow a precise determination of the effective properties of the material samples to be investigated, as previously shown for ceria–zirconia powders [16]. If these samples consist of several components, as is the case with porous and soot-loaded filter samples, the properties of the mixture components can be determined using mixing rules. In this work, the mixing rules established in [15] were used to derive the dielectric soot properties.

### 2.2. RF-Based Filter Monitoring

Measurements on filter substrates installed in an exhaust do not allow for the use of the MCP, since their canning, which forms the resonator cavity, is largely filled by the filter. However, the RF sensor can still use the general correlation between the shift in the resonant parameters and the dielectric properties of the sample [11]. Therefore, it is sufficient to examine the measured resonant parameters alone to diagnose the state of an exhaust gas after treatment system [8]. In the case of particulate filters, the evaluation of resonant modes is only possible for samples with no or only low soot loading [17]. Due to the increasing attenuation of the |*S*_21_| signal with the soot load, however, it is still possible to monitor the filter condition without an evaluable resonant mode. For this purpose, the |*S*_21_| signal averaged over a wide frequency range has usually been considered in the literature [9,18,19]. Previous work on DPFs has already shown that the loading detection is strongly influenced by the filter temperature. Changes in the exhaust gas humidity also have a measurable effect on the RF signal, although it is significantly weaker than those of soot and temperature [20]. For applications of the RF sensor for GPFs, an additional influence due to the soot type could be found, which will be investigated in more detail in this work [9].

## 3. Methodology for Evaluating the RF Properties of Soot

### 3.1. Generation of Soot-Loaded Particulate Filters

In this work, soot was deposited solely on ceramic particulate filters with a honeycomb structure. The filters were cordierite substrates supplied by Corning, Inc. (Corning, NY, USA) with a length of 5′′ (127 mm), a diameter of 5.2′′ (132 mm), a cell density of 200 cpsi, and a wall thickness of 8.5 mil (215.9 μm). The filters are designed for use in gasoline engine vehicles and are not catalytically coated. For the lab test bench measurements, bore cores with a diameter of 48 mm were cut out from the filters. The filter volume of the cores was, therefore, 0.23 l. For analyses with the MCP, only powder samples and not entire filter cores can be examined. Therefore, a 15 mm thick slice was taken from the center of the cores and crushed into particles of about 1 mm in size using a mortar. The samples were not ground to avoid a significant change in the local distribution of the soot particles in the substrate pores compared with that of an intact filter. Otherwise, an effect on the RF properties of the soot samples as a result of the grinding process would not be possible to rule out.

To analyze the behavior of the RF sensor under close to real-world conditions, the particulate filters were loaded with soot on an engine test bench. In order to simulate different operating conditions that may influence the soot characteristics, the engine was operated with different fuel qualities at different load points and by using multiple electronic control unit (ECU) settings. A direct-injection gasoline engine (VW EA888) with a three-way catalyst (TWC) close to the engine and the characteristics listed in Table 1 was used for all tests. The particulate filters were located 0.7 m downstream of the TWC. The exhaust gas system was modified to allow for a simultaneous loading of four filter cores. Thus, soot samples produced under the same conditions were available for the MCP and RF sensor measurements. The filter cores were installed with a mounting mat in cannings with an inner diameter of 50 mm, which could be used on both the engine and the lab test benches without further modification.

To analyze the influence of the engine load on the soot properties, the filter cores were loaded at two stationary operating points and during a real driving emissions (RDE) test simulated on the engine test bench. Due to the low particulate concentration during the RDE, the particulate filter was loaded over several driving cycles to generate sufficient soot loading for the subsequent tests. For this reason, the filters were also loaded for different durations at the stationary operating points.

The engine load at the stationary operating points corresponded to those when driving a D-segment vehicle at constant speeds of 120 km/h and 160 km/h, respectively. The 120 km/h point is within the load range of a typical RDE cycle, while, at 160 km/h, the engine is operated at rotational speeds that are outside those of a typical driving cycle. It is necessary to consider such operating points in order to provide a reliable diagnosis of the GPF loading state under all possible driving conditions. In the following, the operating point at 120 km/h is referred to as “OP1” and that at 160 km/h as “OP2”.

To additionally analyze the influence of the fuel combustion on the RF sensor, the stationary load points were operated with different ECU settings. By varying the fuel injection, the structure and quantity of the soot particles formed during combustion can be influenced [21,22]. In this work, the air–fuel ratio *λ*, the start of injection (SOI), and the injection pressure were varied. Compared with the standard specifications of the ECU (setting I), the injection parameters for setting II were varied in such a way that the particle size distribution was almost unaffected [17]. At the OP2 operating point, a further, stronger variation in the injection parameters (setting III) was applied. For all varied settings, combustion took place under rich conditions (*λ* < 1). In contrast with the stoichiometric conditions of the standard ECU settings, this led to an increased formation of soot particles and, thus, to a faster loading of the filter.

Another factor influencing particle formation was the used fuel [23,24,25,26,27,28,29]. A higher ethanol content can reduce particle emissions [24] and affect the oxidation kinetics [25]. In addition, the aromatic compounds in the fuel not only have an impact on the amount of VOCs [26], but also on the total mass of the formed particles [27]. This influence is mainly due to long-chain aromatics (C9/C9+) [28,29]. To determine whether the fuel also influenced the dielectric properties of the soot, the engine test bench was operated with different fuels. In addition to conventional gas station fuel Super E05, a reference fuel used for certification (CEC Legislative Fuel RF-02-08 E5) and a low-quality fuel with properties outside the permitted tolerances of Super E05, both supplied by Haltermann Carless, were used. The latter has an increased content of long-chain aromatics, while the reference fuel is between the two other fuels with respect to this parameter. In the following, the sample identification is provided by a combination of the operating point, the ECU setting, and the fuel, whereby Super E05 is abbreviated as “S”, the reference fuel as “R” and the low-quality fuel as “L”. For example, OP1-II-S indicates the soot produced at 120 km/h with ECU setting II and Super E05 fuel.

In addition to the soot produced on the engine test bench, the synthetic soot PrintexU, which is often used as a surrogate for gasoline soot in automotive applications [30,31], was also evaluated regarding its effect on the RF sensor for comparison purposes. For this purpose, filter cores were loaded with PrintexU by fluidizing the particles and transporting them into the filter using a nitrogen carrier gas stream.

**Figure 1:**
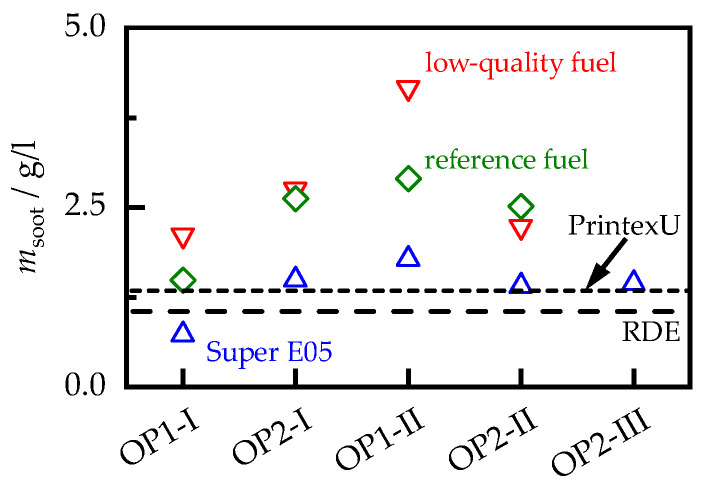
Soot loading $m_{\mathrm{soot}}$ of the filter samples. The values for the PrintexU and RDE samples are shown as dashed lines.

To determine the soot properties, the soot loading $m_{\mathrm{soot}}$ of all samples must be known. In this work, this is provided in relation to the filter volume and was determined by comparing the filter mass before and after the oxidation of the entire soot. The oxidation was carried out in a chamber furnace at 700 °C under ambient air conditions. All weighing was conducted on the downstream half of the filter cores, as the other half was destroyed to prepare the samples for the MCP measurements. Differences in loading between the front and back sections of a filter, and, therefore, a deviation from weighing a whole filter core, are not to be expected, since there is usually a uniform soot distribution in particulate filters [32,33]. The cores were weighed using a Kern PLJ 2000-3A precision balance, which has a linearity error of, at most, 4 mg. This corresponds to an uncertainty in the loading determination of up to 0.035 g/L and can, therefore, lead to uncertainties in the measured RF properties of up to 5% for the sample with the lowest soot loading (OP1-I-S). To reduce the influence of humidity, the samples were stored in a drying oven at 120 °C for at least 24 h prior to weighing. Using these methods, the soot loadings $m_{\mathrm{soot}}$ shown in Figure 1 were determined. As the samples were loaded over different durations, it was not possible to conclude from $m_{\mathrm{soot}}$ to the soot particle concentration produced by the engine.

### 3.2. Examination of the Crushed Soot-Loaded Filter Samples Using the MCP

**Figure 2:**
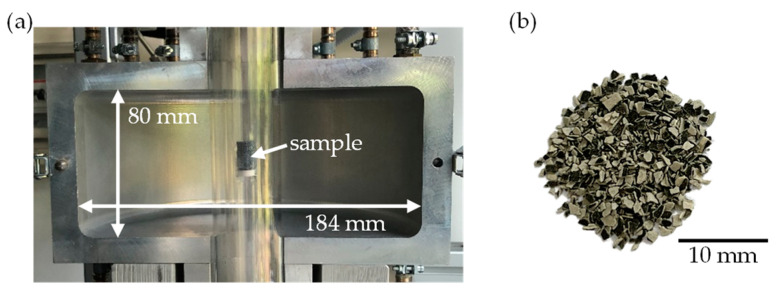
(**a**) Photograph of the MCP resonator with an indication of the dimensions of the resonance cavity; (**b**) Photograph of a crushed soot-loaded particulate filter, which was used for the measurements in the MCP resonator.

The dielectric properties of the soot samples were determined on the crushed filter cores (Figure 2b) using the MCP method described previously. The resonator used for this purpose has already been used in previous studies [34] to characterize powder materials. It allows for the measurement of the filter samples under different gas atmospheres at temperatures of up to 600 °C. A detailed description of the resonator setup (Figure 2a) can be found in [35].

The samples were flushed with an adjustable gas atmosphere at a total flow rate of 500 mL/min. Lean exhaust gas conditions were created by adding oxygen (O_2_) to the nitrogen (N_2_) carrier gas. To determine the parameters of the resonant mode TM_010_ (located at a frequency of approximately 1.18 GHz), the S-parameter S_21_ was measured using a vector network analyzer (VNA, Anritsu MS46322B). Prior to measuring each material sample, a calibration measurement was performed with an empty sample tube to calculate the dielectric material properties from the resonant parameter shift using the MCP. To compare the measurements with and without the sample at the same sample temperature *T*_GPF_, it was determined via the mean value of two type-K thermocouples located inside the sample tube, but outside the resonator cavity. Due to the heatability and the adjustable gas atmosphere, the soot particles deposited on the filter substrate could be removed by oxidation during measurement. This made it possible to analyze the RF properties of the same filter sample in both the soot-loaded and soot-free states. Before and after soot removal, the dielectric properties of the samples were determined at several temperature levels ranging from 20 to 600 °C in a nitrogen atmosphere to prevent oxidation.

To derive the dielectric properties of the soot from these measurement data, the influence of the air content of the porous sample was first removed using the mixing rule determined in [15]. The soot properties could then be derived from the difference between the properties of the soot-loaded samples and the soot-free filter substrate using the second mixing rule described in [15]. To account for the influence of air and cordierite content using the mixing rules, the densities of the filter substrate and the soot must be known. Therefore, the particle volume was measured using a gas pycnometer (Micromeritics AccuPyc 1330). For the cordierite-based filter substrate, a density of 2504 mg/cm^3^ was determined. Due to the small amount of engine soot available, it was not possible to measure the density of the soot particles. Instead, the particle density of the synthetic soot PrintexU, which was measured to be 1060 kg/m^3^, was used for the application of the mixing rule for all samples. This is in agreement with the values determined in [36,37,38] for engine soot of the same particle diameter.

### 3.3. Examination of the Filter Cores on a Lab Test Bench

**Figure 3:**
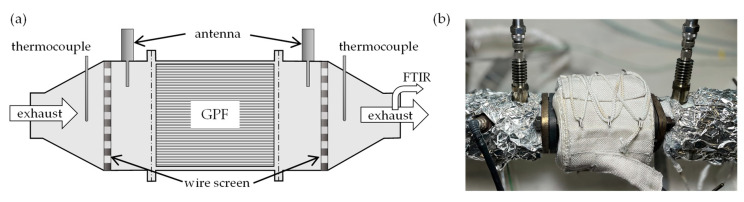
(**a**) Schematic illustration of the measurement setup for the evaluation of the filter cores on the lab test bench. (**b**) Photograph of the measurement setup with the filter canning in the center of the picture enveloped by a heating element.

The filter cores with a diameter of 48 mm were analyzed on a lab test bench. Here, the GPFs could be installed together with the canning already used on the engine test bench. The measurement setup is shown schematically in Figure 3a) and in a photograph in Figure 3b). The cylindrical resonator geometry with a length of 295 mm was defined by the wire screens installed upstream and downstream of the filter. The coaxial antennas required to measure the response of the RF sensor were located in adapter pieces connected to the canning. While the outer conductor was aligned with the inside of the canning, the inner conductor extended 25 mm into the canning. The scattering parameters were recorded using a vector network analyzer (VNA, Anritsu MS46322A, Atsugi, Kanagawa Prefecture, Japan) connected to the antennas. A synthetic exhaust gas flow of 40 L/min with nitrogen as carrier gas could be created via mass flow controllers. A close-to-real-world humidity of up to 10% could be established. Before passing through the GPF, the exhaust gas could be heated to up to 600 °C by an in-line heater. Heat losses through the outer walls of the cannings were reduced by heating elements installed there. This resulted in a maximum temperature gradient of 5 K between the thermocouples located outside of the resonator geometry during stationary operation. Owing to this small difference, the filter temperature *T*_GPF_ was defined as the mean value of the temperatures measured by the thermocouples. After the filter, a constant gas flow of 1 l/min was pumped through a Fourier-Transform Infrared spectrometer (FTIR, MKS Multigas 2030) for gas analysis.

**Figure 4:**
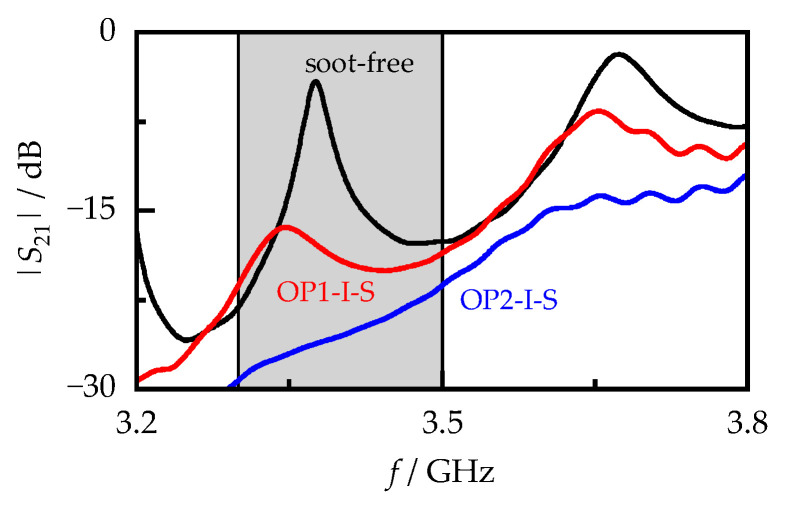
Transmission signal |*S*_21_| of one soot-free and two soot-loaded (OP1-I-S and OP2-I-S) filter cores at 400 °C over the frequency. The |*S*_21_| signal averaged over the gray-shaded frequency range from 3.3 to 3.5 GHz was used as the signal of the RF sensor.

Figure 4 shows the spectra of selected filter samples at a temperature of 400 °C. While the maximum of a resonant mode was still clearly recognizable for the soot-free filter and for the OP1-I-S sample with a soot loading of 0.73 g/L, it disappeared at higher loadings (OP2-I-S with 1.49 g/L soot) due to the stronger signal attenuation. Therefore, the resonant parameters ($f_{\mathrm{res}}$ and $Q_{0}^{-1}$) could not be used to determine the soot-dependent behavior of the RF sensor. Instead, the averaged transmission signal $S_{21,m}$ in the frequency range from 3.3 to 3.5 GHz was used (Equation (4)). This range was chosen because it showed the highest sensitivity in preliminary measurements due to overlap with the TE_112_ resonant mode located at around 3.4 GHz, which was strongly attenuated by the soot loading [17].

(4) $$S_{21,m}=\overset{3.5\;\mathrm{GHz}}{\underset{3.3\;\mathrm{GHz}}{\sum }}20\cdot \log_{10}\left|S_{21}\left(f\right)\right|$$

In [17], it was already shown that the RF signal $S_{21,m}$ changed linearly with the soot loading of the particulate filter. Deviations from this linearity can occur, especially with high soot loadings and, thus, a strongly attenuated signal. In [9], for example, simulations showed a drop in sensitivity at attenuations of over −40 dB. Such attenuation values did not occur in any of the samples measured in this work, with the exception of the sample with the highest soot loading, OP1-II-L. In addition, following the measurements carried out in chapter 5, the stored soot was burned off on the lab test bench. For all samples, $S_{21,m}$ showed a linear correlation with the oxidized soot mass calculated on the basis of the emitted CO and CO_2_ concentrations measured by the FTIR. Therefore, the sensitivity of the RF sensor to soot $s_{\mathrm{soot}}$ could be determined using Equation (5).

(5) $$s_{\mathrm{soot}}=\frac{S_{21,m}-S_{21,m}{(m}_{\mathrm{soot}}=0\;g/L)}{m_{\mathrm{soot}}}$$

The filter cores were examined in the same temperature range as the crushed filter samples by heating them up in several steps from room temperature up to 600 °C. At each temperature step above 100 °C, after reaching a constant temperature, the synthetic exhaust gas was humidified with an absolute humidity $c_{H_{2}O}$ of up to 10 vol.% to evaluate the influence of humidity on the RF sensor.

## 4. RF Properties of Filter Substrate

**Figure 5:**
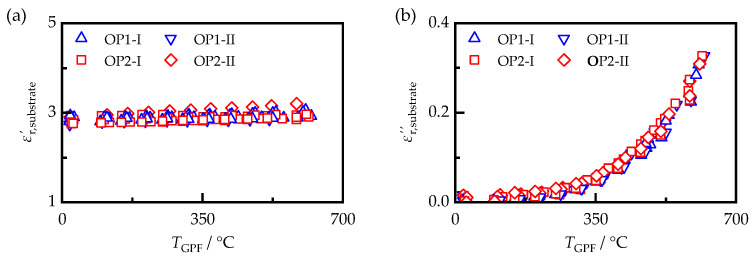
(**a**) Permittivity $\varepsilon_{r,\mathrm{substrate}}^{\prime }$ and (**b**) dielectric losses $\varepsilon_{r,\mathrm{substrate}}^{\prime \prime }$ of the filter substrate of multiple samples measured by the MCP as a function of sample temperature $T_{\mathrm{GPF}}$.

To calculate the soot properties, it is necessary to know the dielectric properties of the filter substrate. They also need to be known in order to allow a simulative description of the soot detection behavior of the RF sensor, as in [39]. For gasoline particulate filters, the substrate material is usually cordierite. Using the MCP, the dielectric properties of the filter samples used in this work could be determined after the deposited soot was removed by oxidation. The resulting dielectric properties of all samples generated at ECU settings I and II are shown in Figure 5.

Between the analyzed samples, which were obtained from different substrates of the same filter type, there were only minimal differences in both permittivity and dielectric losses. For example, for the permittivity, the standard deviation between samples of the same substrate material was less than 5% of the measured value of $\varepsilon_{r,\mathrm{substrate}}^{\prime }$. The permittivity increased slightly with temperature and could be described on average for all samples by a linear dependence according to Equation (6).

(6) $$\varepsilon_{r,\mathrm{substrate}}^{\prime }=2.83+1.95\cdot 10^{-4}\cdot T_{\mathrm{GPF}}/{}^{\circ}C$$

The dielectric losses $\varepsilon_{r,\mathrm{substrate}}^{\prime \prime }$, as typical for ceramic materials, are thermally activated. While $\varepsilon_{r,\mathrm{substrate}}^{\prime \prime }$ is less than 0.02 at room temperature, it increases to more than 0.3 at temperatures above 600 °C. Since, in the literature, the material conductivity is usually used to describe the electrical behavior of ceramics, the substrate conductivity $\sigma_{\mathrm{substrate}}$ was derived from the dielectric losses. This was possible by assuming that the conductivity exceeded the polarization losses significantly ($\varepsilon_{r,\mathrm{pol}}^{\prime \prime }$ << ${\sigma /(\varepsilon }_{0\;}\omega )$). Equation (1) could then be used to derive $\sigma_{\mathrm{substrate}}$, taking into account the frequency of the examined resonant mode of about 1.18 GHz. For a thermally activated conductivity, the temperature-dependent behavior could be described by the Arrhenius equation (Equation (7)) using two parameters—the pre-exponential factor $\sigma_{0}$ and the activation energy $E_{A}$ [40]. In this equation, $k_{B}$ represents the Boltzmann constant and *T* is the substrate temperature. By fitting a linear regression line to the measured data in the Arrhenius plot, an activation energy $E_{A}$ of 0.32 eV and a standard deviation of 0.015 eV could be determined from its slope. The associated pre-exponential factor $\sigma_{0}$ was 1.18 S/m.

(7) $$\sigma_{\mathrm{substrate}}=\sigma_{0}\cdot e^{-\frac{E_{A}}{k_{B}\cdot T}}$$

There are only a few studies in the literature on the dielectric properties of cordierite that can provide reference values. In addition, cordierite is usually investigated in its powder form and not after it has been processed into a monolithic filter. In [41], a permittivity $\varepsilon_{r}^{\prime }$ of 6.2 and dielectric losses $\varepsilon_{r}^{\prime \prime }$ of 0.05 were found for cordierite powder. The measurements were performed at lower frequencies (<1 MHz) than the operating frequency of the RF sensor, while the complex permittivity decreased with increasing measurement frequency. In the same frequency range, Ref. [42] found, for dense cordierite-based glass–ceramic composites, values for $\varepsilon_{r}^{\prime }$ of about 4.4 and dielectric losses of no more than 0.02 at room temperature. In [43], a higher $\varepsilon_{r}^{\prime }$ of 4.6 compared with the MCP measurements was also determined at a higher frequency range of about 19 GHz. In addition, this study was performed at room temperature only and did not include measurements of dielectric losses. Also, at microwave frequencies at 17.54 GHz, a permittivity between 4.6 and 5.3 was measured in [44], depending on the material composition. However, all of the mentioned literature refers to cordierite samples with low porosity (<5%). Therefore, it is unclear whether these results can be transferred to the porous samples investigated in this work (porosity of about 80%), since, for the determination of the bulk permittivity, a mixing rule, which can strongly affect the absolute value of the bulk properties, had to be applied to account for the air content. Furthermore, since the exact chemical composition of the filter substrates used in this work was not known, and the literature data show significant differences between different cordierite samples, no further investigations were made to determine the cause of these differences.

Regarding the temperature-dependent conductivity, as for the permittivity, there are only a few comparative values in the literature, which are also limited to considerations of direct current (DC) conductivity. In [45], an activation energy of 0.8 eV was determined for a signal crystal of cordierite. For polycrystalline cordierite, a value of 1.0 eV was determined in [46]. Whether these results can be transferred to the radio frequency range and are thus comparable to the results obtained in this work is, however, questionable due to the frequency dependence of loss mechanisms.

**Figure 6:**
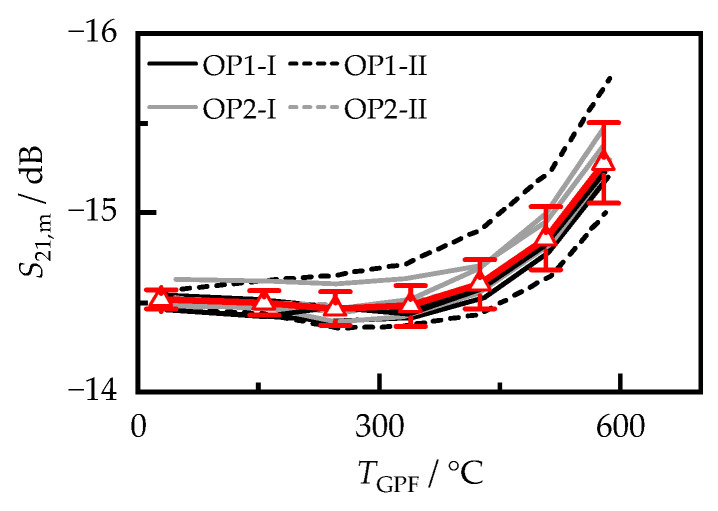
$S_{21,m}$ of the filter cores loaded on the engine test bench after soot removal by oxidation. The red curve shows the average of all measurements, including the standard deviation.

In addition to the dielectric properties of cordierite, the temperature-dependent signal of the RF sensor was also determined after the soot was removed from the filter samples by oxidation. Knowledge of this is necessary to calculate the sensitivity of the RF sensor $s_{\mathrm{soot}}$ according to Equation (5). The RF sensor signal is, therefore, not only affected by the filter properties, but also by other factors, such as the exact geometry of the bore core and canning or the properties of the mounting mat. Nevertheless, the temperature-dependent RF signals of the investigated filter cores varied only slightly (Figure 6). The largest deviations occurred in the OP1-II samples of the reference and the low-quality fuels. Although the standard deviation of the RF signal increased with increasing temperature, it was still less than 0.25 dB at almost 600 °C. Such a change in attenuation could already be caused by the deposition of less than 50 mg/L engine soot [17]. Thus, under realistic sensor operation conditions, the differences between the samples would be negligible, even at low soot loading. In the following chapter, the sensitivity of the RF sensor $s_{\mathrm{soot}}$ is determined for the individual samples in relation to the respective soot-free condition of the same filter. For samples OP1-II-S and OP2-II-S, however, no filter cores were available for the soot oxidation and thus for the determination of the soot-free sensor signal, which is the reason why the mean signal shown in Figure 6 was used for the further evaluation of these samples. Assuming its maximum standard deviation and the sensor signal of these samples present in the soot-loaded state, this would disturb the determined value of $s_{\mathrm{soot}}$ by a maximum of only 3%.

## 5. RF Properties of Soot

### 5.1. Dielectric Properties of Soot

**Figure 7:**
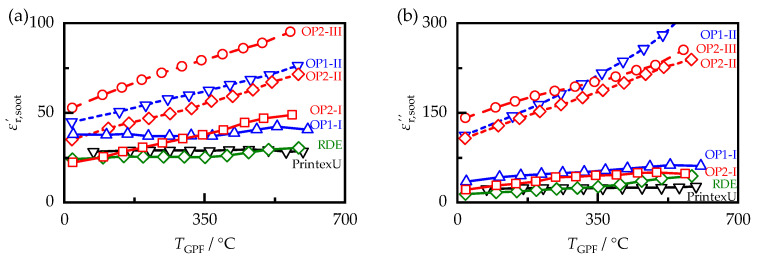
(**a**) Permittivity $\varepsilon_{r,\mathrm{soot}}^{\prime }$ and (**b**) dielectric losses $\varepsilon_{r,\mathrm{soot}}^{\prime \prime }$ of PrintexU, as well as soot samples generated with Super E05 fuel, depending on sample temperature $T_{\mathrm{GPF}}$.

With the dielectric properties of the filter substrate now known, the soot properties can be derived from the effective sample properties determined with the MCP using the mixing rules determined in [15] for mixtures of filter substrate and synthetic soot. Due to the measurement uncertainties observed in Section 4 and the possible inaccuracy in the determination of the soot mass, deviations in the determined dielectric properties of up to 10% cannot be ruled out. Figure 7 shows the dielectric soot properties of the samples produced with Super E05 fuel and of the synthetic soot PrintexU, as it depends on the sample temperature $T_{\mathrm{GPF}}$. In the frequency range investigated in this work, reference values in the literature are mainly found for synthetic soot. In [47], permittivities with values between 60 and 180 were determined for various synthetic carbon blacks with a graphite structure at a frequency of 1 GHz and room temperature, which are higher than those of PrintexU (at room temperature, $\varepsilon_{r,\mathrm{soot}}^{\prime }$ has a value of 28 and $\varepsilon_{r,\mathrm{soot}}^{\prime \prime }$ of 23), which was examined in this work. However, the dielectric losses with values between 20 and 60 also measured agree with the properties of PrintexU as determined here. The properties determined in [48] for porous samples of synthetic soot are in the range of the values measured in this work, with a permittivity of 60 and dielectric losses of 50, assuming the same mixing rules as those used in this work. Also, using this mixing rule, for a sample with a 25% synthetic carbon black content measured in [49], a permittivity of 38 and dielectric losses of 16 at a frequency of 2 GHz could be derived for the bulk properties, which are also in the range of the values determined in this work.

The values of the permittivity, as well as the losses of all soot samples, clearly exceeded those of the filter substrate. Furthermore, they varied significantly between the different soot samples. For example, at room temperature, the permittivities $\varepsilon_{r,\mathrm{soot}}^{\prime }$ differed by a factor of up to 2.4 and the dielectric losses $\varepsilon_{r,\mathrm{soot}}^{\prime \prime }$ by up to 10.5 times. Although the dielectric properties of all samples increased with increasing temperature, this effect varied depending on the type of soot. Therefore, the differences between the samples increased with temperature.

**Figure 8:**
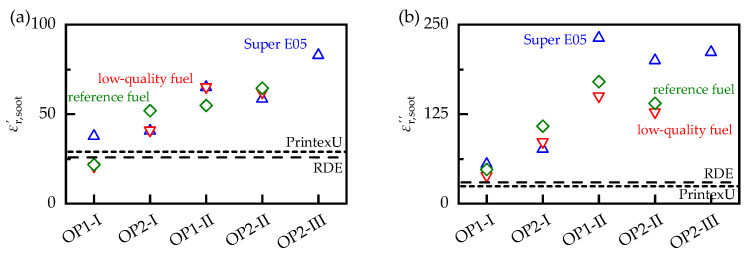
(**a**) Permittivity $\varepsilon_{r,\mathrm{soot}}^{\prime }$ and (**b**) dielectric losses $\varepsilon_{r,\mathrm{soot}}^{\prime \prime }$ of different soot samples at a temperature $T_{\mathrm{GPF}}$ of 400 °C.

For a more in-depth evaluation of the influences acting on the soot samples, their properties were evaluated at a temperature of 400 °C, which is a typical operating temperature for gasoline particulate filters [50]. Since the steady-state temperatures at which the soot samples were analyzed varied slightly from sample to sample and were not exactly 400 °C, the dielectric properties at this temperature were determined by interpolating the adjacent measured values using a cubic spline. The resulting dielectric properties $\varepsilon_{r,\mathrm{soot}}^{\prime }$ and $\varepsilon_{r,\mathrm{soot}}^{\prime \prime }$ are shown in Figure 8 for all examined soot samples.

The values of the synthetic soot PrintexU matched those of the soot particles occurring during real driving cycles (RDE) and were only slightly lower than those of the OP1-I soot in terms of losses. However, its permittivity and losses were significantly lower than those of most other soot samples. Engine operation under rich exhaust gas conditions at ECU setting II resulted in a significant increase of 73% in permittivity and 147% in losses compared with the stoichiometric exhaust gas composition at setting I. Further adjustments of the engine settings from OP2-II-S to OP2-III-S resulted in an additional increase in the dielectric properties of 42% respectively 5%. However, these values were only of limited significance, as they were based solely on soot samples generated under load-point OP2 with Super E05 fuel. Increasing the engine load from OP1 to OP2 also resulted in an increase in the dielectric properties, which was weaker than the influence of the ECU settings, with 20% for $\varepsilon_{r,\mathrm{soot}}^{\prime }$ and 6.5% for $\varepsilon_{r,\mathrm{soot}}^{\prime \prime }$. This is mainly due to the low dependency of the samples generated under setting II. Considering only the stoichiometric engine operation at setting I, the increase due to the load change increased to 66% respectively 91%, and was thus only slightly below the ECU influence. Compared with the ECU and load point influence, the fuel influence was much smaller, with a standard deviation of about 13% for $\varepsilon_{r,\mathrm{soot}}^{\prime }$ and 22% for $\varepsilon_{r,\mathrm{soot}}^{\prime \prime }$ relative to the mean value of the respective operating points. It also shows no clear trend toward higher or lower dielectric properties. For an automotive application of the RF sensor, however, this influence, as well as the influence of the load point and the ECU settings, would have to be corrected to allow an accurate determination of the soot loading.

### 5.2. Signal of the RF Sensor

**Figure 9:**
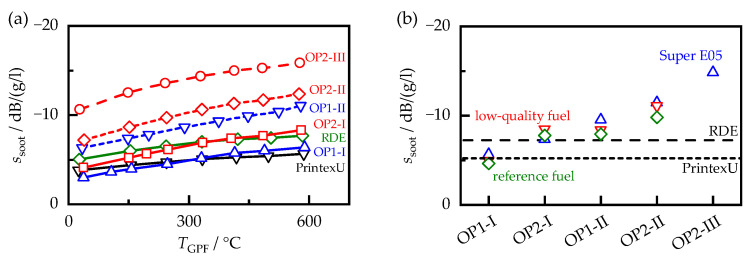
(**a**) Sensitivity of the RF sensor $s_{\mathrm{soot}}$ for filters loaded with PrintexU and with engine soot generated with Super E05 fuel over the filter temperature. (**b**) Sensitivity of the RF sensor $s_{\mathrm{soot}}$ for different soot samples at a temperature $T_{\mathrm{GPF}}$ of 400 °C.

To determine if the sensitivity of the RF sensor to soot loading $s_{\mathrm{soot}}$ could be directly derived from the dielectric properties of soot, the sensor sensitivity was determined using Equation (5) for all soot-loaded filters on the lab test bench in the same temperature range as that for the MCP measurements. For the samples produced with Super E05 fuel and for the synthetic soot PrintexU, $s_{\mathrm{soot}}$ is shown as a function of the sample temperature $T_{\mathrm{GPF}}$ in Figure 9a). Similar to the dielectric properties, $s_{\mathrm{soot}}$ also increased with temperature. In addition, the RF sensor reacted with different sensitivities to the soot samples. This was consistent with the described load point and ECU setting’s influence on the dielectric soot properties. At room temperature, there was a factor of 3.5 between the highest and lowest sensor sensitivities, which was larger than the spread between the permittivities of the different soot samples $\varepsilon_{r,\mathrm{soot}}^{\prime }$, but smaller than the spread between the dielectric losses $\varepsilon_{r,\mathrm{soot}}^{\prime \prime }$. Compared with the effect of engine operation on the RF sensor, changes in filter temperature had a much smaller effect on the sensor sensitivity. For example, even for the soot sample with the largest temperature effect, an uncompensated change in the filter temperature of 20 K would alter $s_{\mathrm{soot}}$ by only 5%.

To compare the soot samples more easily, the sensor sensitivity was evaluated at a temperature of 400 °C, similar to the dielectric properties, by interpolation between the adjacent measured values using a cubic spline (Figure 9b). In general, the same systematic correlations existed between the soot samples generated on the engine test bench as those for the dielectric properties. Switching from ECU setting I to II resulted in a 50% increase in $s_{\mathrm{soot}}$, which was also observed for the dielectric properties, albeit to a greater extent (73% in $\varepsilon_{r,\mathrm{soot}}^{\prime }$ and 147% in $\varepsilon_{r,\mathrm{soot}}^{\prime \prime }$). The increase in engine load from OP1 to OP2 also resulted in a different increase in sensitivity, depending on the ECU setting. At setting I, the load change resulted in a 54% higher sensitivity, whereas at setting II, it only led to a 25% higher sensitivity. The fuel influence had, again, a smaller effect on the sensor sensitivity than the ECU and load influence, with a standard deviation of about 9% compared with the mean value of the respective operating points.

**Figure 10:**
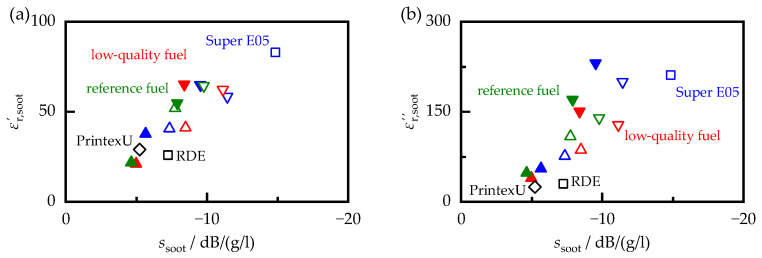
Permittivity $\varepsilon_{r,\mathrm{soot}}^{\prime }$ (**a**) and dielectric losses $\varepsilon_{r,\mathrm{soot}}^{\prime \prime }$ (**b**) versus the sensitivity of the RF sensor $s_{\mathrm{soot}}$. Filled symbols correspond to OP1, open symbols correspond to OP2. ECU setting I is represented by upward-pointing triangles, II by downward-pointing triangles, and III by a square. The PrintexU and RDE samples are labeled separately.

While the overall trends in the MCP and filter core measurements were similar, those of individual samples differed significantly. For example, the dielectric properties of PrintexU and the soot produced during the RDE cycle were almost identical. On the other hand, the $s_{\mathrm{soot}}$ of PrintexU was almost 30% lower than that of the RDE soot. This effect became apparent by plotting the dielectric properties against the sensitivity of the RF sensor in Figure 10. In principle, this showed a linear relationship. However, a direct determination of the sensitivity of the RF sensor from the permittivity or dielectric losses of the soot alone, without taking into account other influencing factors, would only be possible with uncertainty. One reason for this may be the fact that both components of the dielectric properties affected the frequency spectrum that served as the signal for the RF sensor. Another reason can be found in the different measuring frequencies of the two systems (MCP worked at 1.18 GHz, while for the determination of $s_{\mathrm{soot}}$, a higher frequency range of 3.3 to 3.5 GHz was used). For example, in [49], a slight, but linear, frequency dependence of the dielectric properties was found for carbon black, with higher frequencies leading to a decrease in the complex permittivity.

## 6. Method for Correcting the Engine Influence on the RF Sensor

The correct monitoring of the filter loading requires a correction of the soot’s influence on the RF sensor. One option is to calibrate the RF sensor for all engine operating conditions. The effective sensor sensitivity resulting from the mixture of the different types of soot could then be determined by weighing the different operating conditions by time [51]. However, this would require extensive load measurements on the engine test bench, including a recalibration for each canning and filter geometry. For this reason, it is essential to be able to determine the sensor sensitivity during driving conditions. Therefore, easily detectable cross-influences on the RF sensor were examined for a correlation with the sensor sensitivity $s_{\mathrm{soot}}$. Although there was no direct correlation with the temperature influence, it may be possible to correct for $s_{\mathrm{soot}}$ by considering the signal changes caused by variations in exhaust gas humidity.

### 6.1. Influcence of Humidity on the RF Sensor

**Figure 11:**
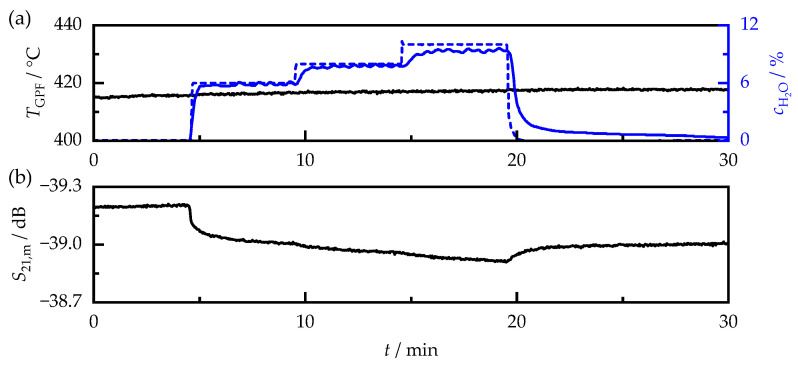
Effect of the exhaust gas humidity variation on the RF sensor signal over time when measuring the OP2-II-R sample with a soot loading of 2.51 g/L. (**a**) Filter temperature $T_{\mathrm{GPF}}$ (black) and exhaust gas humidity $c_{H_{2}O}$ set on the lab test bench (dashed blue line) and measured downstream of the filter by FTIR (solid blue line). (**b**) $S_{21,m}$ signal during humidity variation.

The exhaust gas humidity can have a significant effect on the RF sensor signal in catalytic converter systems [52]. However, for particulate filters, a negligible influence has been found compared with the sensitivity to temperature and soot mass [20]. Nevertheless, a change in the RF signal can still be detected, especially in the case of large changes in humidity, e.g., due to fuel cutoffs, as the humidity drops to the ambient air value. To quantify the influence of humidity, the variation in the exhaust gas humidity $c_{H_{2}O}$ (shown exemplarily in Figure 11) was carried out for all temperatures above 100 °C. The previously dry exhaust gas was exposed to absolute humidities $c_{H_{2}O}$ of 6, 8, and 10 vol.% for five minutes each, covering the relevant range of conditions occurring during real driving operation.

For the OP2-II-R sample shown in Figure 11, there was a decrease in signal attenuation due to the humidification of the exhaust gas, which amounted to a maximum of 0.3 dB compared with that of the dry exhaust gas. Considering a soot sensitivity for the OP2-II-R sample of −9.8 dB/(g/L), this corresponded to a change in soot loading of only 0.03 g/L. Similarly, for all other soot samples, the addition of water had only a small effect on the RF signal. Beyond that, a hysteresis of the attenuation signal could be observed for some samples during exhaust gas humidification. Even after the prolonged purging of the filter sample with dry exhaust gas, $S_{21,m}$ remained at a lower attenuation than that before the humidity variation. At the same time, the release of small amounts of CO (<1 ppm) could be detected, indicating that the hysteresis may not have been due to a change in the dielectric properties of the soot itself, but rather to a slight oxidation of the stored soot.

The soot-free filter substrates showed no signal change due to the exhaust gas humidification. For this reason, the signal shift was evaluated according to Equation (8) in relation to the sensor signal of the humidity- and soot-free filter. The resulting parameter $\theta_{H_{2}O}$ described the relative change in the RF sensor sensitivity $s_{\mathrm{soot}}$ and could be determined without knowledge of the soot loading. To evaluate this parameter, the sensor signal was averaged over one minute prior to the subsequent change in humidity. For the correct determination of humidity’s influence on the filter cores, even the small temperature changes occurring during the measurement must be compensated for. To account for the temperature dependency of $S_{21,m}$, a third-degree polynomial was fitted to the previously determined temperature-dependent RF signal. This was then used to determine the theoretically humidity-free signal $S_{21,m}{(0\;\%}_{H_{2}O})$.

(8) $$\theta_{H_{2}O}=\frac{S_{21,m}{(c}_{H_{2}O})-S_{21,m}{(0\%}_{H_{2}O})}{S_{21,m}{(0\;\%}_{H_{2}O})-S_{21,m}{(0\;\%}_{H_{2}O}{,\;m}_{\mathrm{soot}}=0\;g/L)}\cdot \frac{1}{c_{H_{2}O}}$$

**Figure 12:**
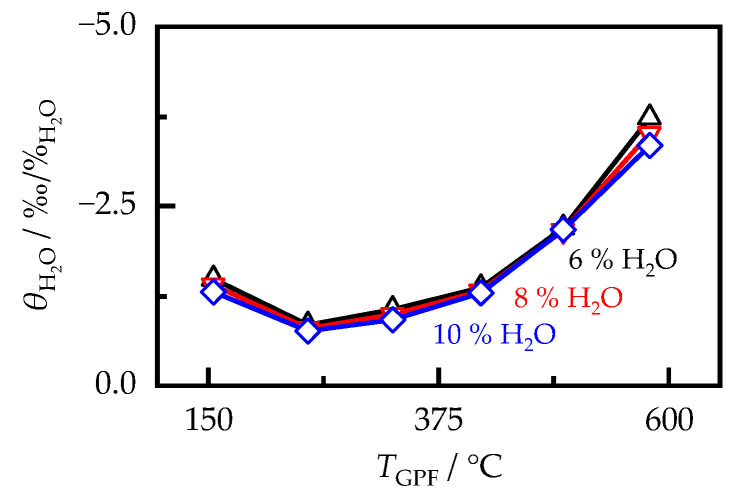
Humidity sensitivity $\theta_{H_{2}O}$ of the RF sensor signal as a function of the filter temperature $T_{\mathrm{GPF}}$ for different exhaust gas humidities $c_{H_{2}O}$ in the case of the OP2-II-R sample with a soot loading of 2.51 g/L.

Before comparing the different soot samples, the general influence of the humidity concentration $c_{H_{2}O}$ on the relative sensitivity $\theta_{H_{2}O}$ was analyzed. As can be seen in Figure 12, exemplarily for the OP2-II-R sample, the three humidity levels resulted in an almost identical relative signal change, independent of the filter temperature. This was also the case for the other soot samples. Therefore, the resulting change in the attenuation signal was linearly related to the exhaust gas humidity and $\theta_{H_{2}O}$ could be considered as independent of $c_{H_{2}O}$. For this reason, the RF signal was subsequently evaluated only at a humidity content of 8 vol.%.

**Figure 13:**
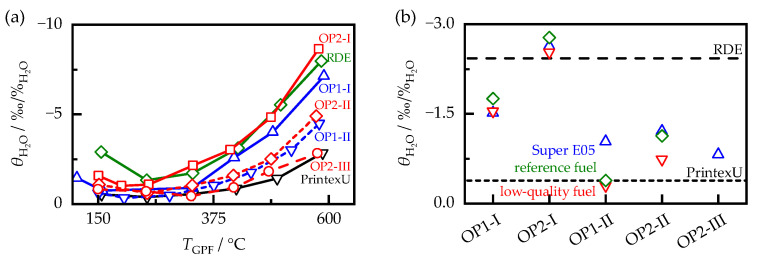
(**a**) Humidity sensitivity $\theta_{H_{2}O}$ of RF sensor signal for filter loaded with PrintexU and with engine soot generated with Super E05 fuel over the filter temperature. (**b**) Humidity sensitivity $\theta_{H_{2}O}$ of the RF sensor signal for different soot samples at a filter temperature of 400 °C.

Figure 13a shows the influence of humidity determined according to Equation (8) for the samples produced with Super E05 fuel, as well as that for the synthetic soot PrintexU depending on the sample temperature $T_{\mathrm{GPF}}$. In principle, all soot samples showed a similar behavior. The cross sensitivity to humidity changes was lowest at temperatures around 300 °C. Toward lower temperatures, it increased slightly, but is still at a low level compared with the temperature influence, with a maximum of −4‰/${\%}_{H_{2}O}$ among all soot-loaded samples. This increase could not be explained by the condensation of liquid water, as it can occur during the cold start of an engine, since, in this case, an increase in dielectric losses and, thus, an increase in the signal attenuation $S_{21,m}$ would be expected [52]. Instead, this effect can be attributed to the definition of $\theta_{H_{2}O}$. Owing to the smaller attenuation increase due to soot loading at lower temperatures (see Figure 9a), an absolutely equal change in the RF signal due to humidity variations had a greater effect on the relative sensitivity $\theta_{H_{2}O}$ than that at higher temperatures. Furthermore, $\theta_{H_{2}O}$ increased significantly above 300 °C, as previously observed for diesel soot [20]. This increase varied depending on the soot sample. For example, at a filter temperature of 600 °C, the humidity sensitivity could vary by a factor of 4 between −2.5 and −10‰/${\%}_{H_{2}O}$.

To allow for a direct comparison between the soot samples, $\theta_{H_{2}O}$ was evaluated, similar to the RF sensor sensitivity to soot $s_{\mathrm{soot}}$ before, at a temperature of 400 °C (Figure 13b). On average, the cross-sensitivity was −1.4‰/${\%}_{H_{2}O}$. Compared with the temperature effect on the RF signal, a 1 vol.% change in exhaust gas moisture had a similar effect as a temperature change of only 1 K. As with $s_{\mathrm{soot}}$, the soot samples also showed systematic correlations among each other. Increasing the engine load from OP1 to OP2 resulted, on average, in a 69% increase in sensitivity. Changing the ECU setting from I to II resulted in a reduction in $\theta_{H_{2}O}$ of 62%. At setting III, the influence of humidity was further reduced. Thus, the humidity cross-sensitivity showed a different behavior with respect to the ECU settings to $s_{\mathrm{soot}}$, where an increase in sensitivity was observed. How the soot-dependent sensor sensitivities can be corrected based on this is discussed in the following chapter. Similarly to $s_{\mathrm{soot}}$, the fuel’s influence on the RF sensor was much weaker than the effect of the ECU or load variations, with a standard deviation of about 16% compared with the mean value of the respective operating points.

### 6.2. Correlation between Humidity and the RF Sensor Sensitivity

**Figure 14:**
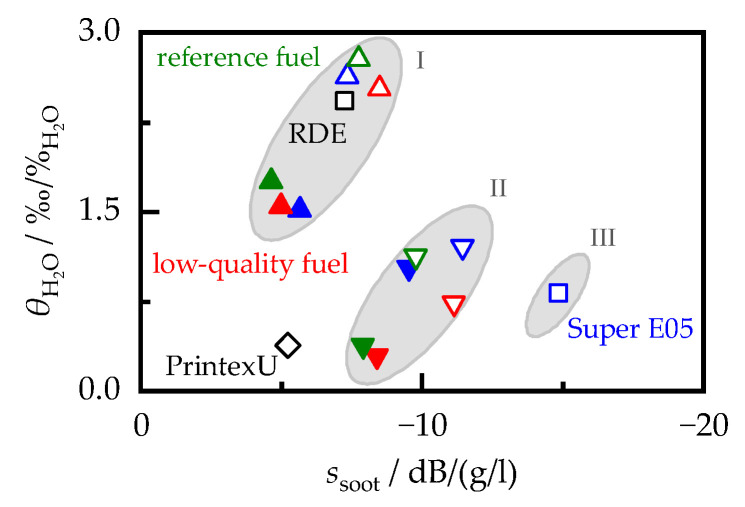
Relative humidity sensitivity $\theta_{H_{2}O}$ plotted against the soot-loading sensitivity of the RF sensor $s_{\mathrm{soot}}$ for all soot samples investigated. Filled symbols correspond to OP1 and open symbols correspond to OP2. ECU setting I is represented by upward-pointing triangles, II by downward-pointing triangles, and III by a square. PrintexU and RDE samples are labeled separately.

For the serial application of the RF sensor in gasoline vehicles, it is necessary to find a method to correct the sensitivity for the influence of the type of soot during driving. The humidity sensitivity $\theta_{H_{2}O}$ can provide a way to determine $s_{\mathrm{soot}}$, since, as shown in Figure 14, soot samples with a higher RF sensor sensitivity are associated with a stronger influence caused by the exhaust gas humidity. This correlation can be explained by the dependency observed in [53] between the adsorption of water on the soot surface and the chemical composition of the soot particles, which, in turn, can affect their dielectric properties. However, the relationship between $\theta_{H_{2}O}$ and $s_{\mathrm{soot}}$ was only valid within the same engine setting. Switching from ECU setting I to II led to an increase in $s_{\mathrm{soot}}$ with a simultaneous decrease in $\theta_{H_{2}O}$. This effect was further enhanced when changing the ECU to setting III. Despite the ECU variation, the general relationship between $s_{\mathrm{soot}}$ and $\theta_{H_{2}O}$ remained unchanged. Therefore, if the ECU settings used during soot formation and the current humidity sensitivity are known, the sensitivity of the RF sensor can be derived. Realistic operation of a gasoline engine primarily uses setting I, which corresponds to standard ECU settings and stoichiometric combustion. Accordingly, the soot generated during the RDE cycle can also be described by the correlation defined by setting I. The humidity sensitivity can be determined in real operation, preferably when the engine operation is switched to a fuel cutoff, as the exhaust gas humidity then changes by several percent in a short period of time. Therefore, for practical application of the RF sensor, it is no longer necessary to calibrate for each individual operating point, but only to analyze a few soot samples to determine their humidity and soot-loading sensitivity.

## 7. Conclusions

The RF sensor provides an alternative to conventional differential pressure sensors for monitoring the soot loading of GPFs. However, previous research has shown that the sensitivity of the RF sensor can be affected by the engine operating conditions during soot formation. This work has now investigated this cross-sensitivity in more detail. In addition, it has been shown that the influence of exhaust gas humidity on the RF sensor can offer a method to correct for the influence of the engine operation.

For this purpose, soot samples were produced on an engine test bench with variations in engine load, ECU settings, and fuel. In order to analyze possible correlations in more detail, the dielectric properties of the soot samples were determined using the microwave cavity perturbation method. Before this could be achieved, the properties of the filter substrate had to be determined. Only minor differences were found between different samples of the same filter type. Measurements with the RF sensor on soot-free GPF confirmed that the changes in the RF signal caused by the different substrates were negligible compared with the signal changes caused by soot loading. Consequently, the RF sensor could be used on different exhaust tracts of the same geometry without additional calibration. The subsequent analysis of the dielectric properties of the generated soot samples showed that both the permittivity and the dielectric losses could vary significantly. This also applied to the sensitivity of the RF sensor determined on filter cores measured in a lab test bench, which could differ depending on the soot by more than a factor of 3. While the fuel properties had only a minor influence on the RF sensor, the engine load and the ECU settings had an equally large effect. Without a correction, the predicted soot mass could, therefore, be inaccurate by a factor of 3. In real operation, however, this would only occur if the vehicle was only driven at one operating point for a longer period of time. Under typical driving conditions, the RF sensor could, therefore, determine the soot loading with a high accuracy, even without correction for the soot type. In [9], for example, an accuracy of ±0.2 g/L was achieved.

However, in order to maintain this accuracy under all possible operating conditions, the influence of the soot type can be corrected by the cross-sensitivity toward exhaust gas humidity. Although it is weak compared with the sensitivity of the RF sensor to soot, or even to other cross-sensitivities, such as the filter temperature, it can be easily evaluated when the engine operation is switched to fuel cut, since this causes a distinct decrease in the exhaust gas water concentration. The resulting change in the RF sensor signal correlates with the engine load during soot formation and is, therefore, suitable as a parameter for estimating the RF sensor sensitivity. However, this correlation is only valid for soot produced within one ECU setting. For an application of the RF sensor, the correlation between the influence of humidity and the sensor sensitivity would have to be determined for each ECU map and thus also for each engine type. In addition, the investigations carried out in this work could not clarify how the influence of the engine load could be predicted. Therefore, further investigations should be carried out on a larger number of soot samples generated at different load points.

## Figures and Tables

**Table 1:** Specifications of the engine test bench.

Number of cylinders	4, in line
Power	125 kW at 3800 min^−1^
Torque	320 Nm at 1500 min^−1^
Displaced volume	1798 cm^3^
Stroke	84.1 mm
Bore	82.5 mm
Compression ratio	9.60:1

## Data Availability

All relevant data presented in the article are stored according to institutional requirements and as such are not available online. However, all data used in this paper can be made available upon request to the authors.

## References

[1] Platt S.M., El Haddad I., Pieber S.M., Zardini A.A., Suarez-Bertoa R., Clairotte M., Daellenbach K.R., Huang R.-J., Slowik J.G., Hellebust S. (2017). Gasoline cars produce more carbonaceous particulate matter than modern filter-equipped diesel cars. Sci. Rep..

[2] European Environment Agency (2020). Air Quality in Europe: 2020 Report.

[3] Lohmann U., Friebel F., Kanji Z.A., Mahrt F., Mensah A.A., Neubauer D. (2020). Future warming exacerbated by aged-soot effect on cloud formation. Nat. Geosci..

[4] Lanzerath P., Wunsch R., Schön C., Bargende M., Reuss H.-C., Wiedemann J. (2017). The first series-production particulate filter for Mercedes-Benz gasoline engines. 17. Internationales Stuttgarter Symposium: Automobil- und Motorentechnik.

[5] Joshi A., Johnson T.V. (2018). Gasoline Particulate Filters—A Review. Emiss. Control Sci. Technol..

[6] Saito C., Nakatani T., Miyairi Y., Yuuki K., Makino M., Kurachi H., Heuss W., Kuki T., Furuta Y., Kattouah P. (2011). New Particulate Filter Concept to Reduce Particle Number Emissions.

[7] Gaiser G., Mucha P. (2004). Prediction of Pressure Drop in Diesel Particulate Filters Considering Ash Deposit and Partial Regenerations.

[8] Dietrich M., Jahn C., Lanzerath P., Moos R. (2015). Microwave-Based Oxidation State and Soot Loading Determination on Gasoline Particulate Filters with Three-Way Catalyst Coating for Homogenously Operated Gasoline Engines. Sensors.

[9] Nicolin P., Boger T., Dietrich M., Haft G., Bachurina A. (2020). Soot Load Monitoring in Gasoline Particulate Filter Applications with RF-Sensors.

[10] Pozar D.M. (2012). Microwave Engineering.

[11] Chen P., Schönebaum S., Simons T., Rauch D., Dietrich M., Moos R., Simon U. (2015). Correlating the Integral Sensing Properties of Zeolites with Molecular Processes by Combining Broadband Impedance and DRIFT Spectroscopy-A New Approach for Bridging the Scales. Sensors.

[12] Chen L. (2005). Microwave Electronics: Measurement and Materials Characterization.

[13] Parkash A., Vaid J.K., Mansingh A. (1979). Measurement of Dielectric Parameters at Microwave Frequencies by Cavity-Perturbation Technique. IEEE Trans. Microw. Theory Tech..

[14] Steiner C., Walter S., Malashchuk V., Hagen G., Kogut I., Fritze H., Moos R. (2020). Determination of the Dielectric Properties of Storage Materials for Exhaust Gas Aftertreatment Using the Microwave Cavity Perturbation Method. Sensors.

[15] Walter S., Schwanzer P., Steiner C., Hagen G., Rabl H.-P., Dietrich M., Moos R. (2022). Mixing Rules for an Exact Determination of the Dielectric Properties of Engine Soot Using the Microwave Cavity Perturbation Method and Its Application in Gasoline Particulate Filters. Sensors.

[16] Steiner C., Hagen G., Kogut I., Fritze H., Moos R. (2022). Analysis of defect chemistry and microstructural effects of non-stoichiometric ceria by the high-temperature microwave cavity perturbation method. J. Eur. Ceram. Soc..

[17] Walter S., Schwanzer P., Hagen G., Haft G., Dietrich M., Rabl H.-P., Moos R., Tille T. (2020). Hochfrequenzsensorik zur direkten Beladungserkennung von Benzinpartikelfiltern. Automobil-Sensorik 3.

[18] Sethia S., Kubinski D., Nerlich H., Naber J. (2020). RF Studies of Soot and Ammonia Loadings on a Combined Particulate Filter and SCR Catalyst. J. Electrochem. Soc..

[19] Sappok A., Ragaller P., Guarino A., Mandelbaum J., Lapenta L., Kolberg D., Newman R., Lu X., Cors D., Bromberg L. (2019). Direct Measurement of Aftertreatment System Stored Water Levels for Improved Dew Point Management Using Radio Frequency Sensing.

[20] Feulner M., Seufert F., Müller A., Hagen G., Moos R. (2017). Influencing Parameters on the Microwave-Based Soot Load Determination of Diesel Particulate Filters. Top. Catal..

[21] Potenza M., Milanese M., de Risi A. (2019). Effect of injection strategies on particulate matter structures of a turbocharged GDI engine. Fuel.

[22] Notheis D., Bertsch M., Velji A., Koch T., Tschöke H., Marohn R. (2017). Untersuchung der Partikelemissionen für unterschiedliche Einspritzstrategien an einem aufgeladenen Ottomotor mit Direkteinspritzung. 10. Tagung Diesel- und Benzindirekteinspritzung 2016.

[23] Kalvakala K.C., Pal P., Aggarwal S.K. (2020). Effects of fuel composition and octane sensitivity on polycyclic aromatic hydrocarbon and soot emissions of gasoline–ethanol blend surrogates. Combust. Flame.

[24] Graves B.M., Koch C.R., Olfert J.S. (2017). Morphology and volatility of particulate matter emitted from a gasoline direct injection engine fuelled on gasoline and ethanol blends. J. Aerosol Sci..

[25] Luo Y., Zhu L., Fang J., Zhuang Z., Guan C., Xia C., Xie X., Huang Z. (2015). Size distribution, chemical composition and oxidation reactivity of particulate matter from gasoline direct injection (GDI) engine fueled with ethanol-gasoline fuel. Appl. Therm. Eng..

[26] Xing J., Shao L., Zheng R., Peng J., Wang W., Guo Q., Wang Y., Qin Y., Shuai S., Hu M. (2017). Individual particles emitted from gasoline engines: Impact of engine types, engine loads and fuel components. J. Clean. Prod..

[27] Manin J., Skeen S., Pickett L., Kurtz E., Anderson J.E. (2014). Effects of Oxygenated Fuels on Combustion and Soot Formation/Oxidation Processes. SAE Int. J. Fuels Lubr..

[28] Yang J., Roth P., Durbin T.D., Karavalakis G. (2019). Impacts of gasoline aromatic and ethanol levels on the emissions from GDI vehicles: Part 1. Influence on regulated and gaseous toxic pollutants. Fuel.

[29] Fatouraie M., Frommherz M., Mosburger M., Chapman E., Li S., McCormick R., Fioroni G. (2018). Investigation of the Impact of Fuel Properties on Particulate Number Emission of a Modern Gasoline Direct Injection Engine.

[30] Boger T., Rose D., Nicolin P., Gunasekaran N., Glasson T. (2015). Oxidation of Soot (Printex^®^ U) in Particulate Filters Operated on Gasoline Engines. Emiss. Control Sci. Technol..

[31] Choi S., Seong H. (2015). Oxidation characteristics of gasoline direct-injection (GDI) engine soot: Catalytic effects of ash and modified kinetic correlation. Combust. Flame.

[32] Huynh C.T., Johnson J.H., Yang S.L., Bagley S.T., Warner J.R. (2003). A One-Dimensional Computational Model for Studying the Filtration and Regeneration Characteristics of a Catalyzed Wall-Flow Diesel Particulate Filter.

[33] Lupše J., Campolo M., Soldati A. (2016). Modelling soot deposition and monolith regeneration for optimal design of automotive DPFs. Chem. Eng. Sci..

[34] Rauch D., Dietrich M., Simons T., Simon U., Porch A., Moos R. (2017). Microwave Cavity Perturbation Studies on H-form and Cu Ion-Exchanged SCR Catalyst Materials: Correlation of Ammonia Storage and Dielectric Properties. Top. Catal..

[35] Dietrich M., Rauch D., Porch A., Moos R. (2014). A Laboratory Test Setup for in Situ Measurements of the Dielectric Properties of Catalyst Powder Samples under Reaction Conditions by Microwave Cavity Perturbation: Set up and Initial Tests. Sensors.

[36] Rissler J., Messing M.E., Malik A.I., Nilsson P.T., Nordin E.Z., Bohgard M., Sanati M., Pagels J.H. (2013). Effective Density Characterization of Soot Agglomerates from Various Sources and Comparison to Aggregation Theory. Aerosol Sci. Technol..

[37] Maricq M., Xu N. (2004). The effective density and fractal dimension of soot particles from premixed flames and motor vehicle exhaust. J. Aerosol Sci..

[38] Momenimovahed A., Olfert J.S. (2015). Effective Density and Volatility of Particles Emitted from Gasoline Direct Injection Vehicles and Implications for Particle Mass Measurement. Aerosol Sci. Technol..

[39] Walter S., Schwanzer P., Hagen G., Haft G., Rabl H.-P., Dietrich M., Moos R. (2020). Modelling the Influence of Different Soot Types on the Radio-Frequency-Based Load Detection of Gasoline Particulate Filters. Sensors.

[40] Kao K.C. (2004). Dielectric Phenomena in Solids.

[41] Camerucci M.A., Urretavizcaya G., Castro M.S., Cavalieri A.L. (2001). Electrical properties and thermal expansion of cordierite and cordierite-mullite materials. J. Eur. Ceram. Soc..

[42] Synkiewicz-Musialska B., Szwagierczak D., Kulawik J., Pałka N., Piasecki P. (2021). Structural, Thermal and Dielectric Properties of Low Dielectric Permittivity Cordierite-Mullite-Glass Substrates at Terahertz Frequencies. Materials.

[43] Ohsato H., Kim J.-S., Kim A.-Y., Cheon C.-I., Chae K.-W. (2011). Millimeter-Wave Dielectric Properties of Cordierite/Indialite Glass Ceramics. Jpn. J. Appl. Phys..

[44] Lou W., Mao M., Song K., Xu K., Liu B., Li W., Yang B., Qi Z., Zhao J., Sun S. (2022). Low permittivity cordierite-based microwave dielectric ceramics for 5G/6G telecommunications. J. Eur. Ceram. Soc..

[45] Schmidbauer E., Mirwald P.W. (1993). Electrical conductivity of cordierite. Mineral. Petrol..

[46] Labrincha J.A., Albuquerque C.M., Ferreira J.M., Ribeiro M.J. (2006). Electrical characterisation of cordierite bodies containing Al-rich anodising sludge. J. Eur. Ceram. Soc..

[47] Atwater J.E., Wheeler J.R.R. (2004). Temperature dependent complex permittivities of graphitized carbon blacks at microwave frequencies between 0.2 and 26 GHz. J. Mater. Sci..

[48] Hotta M., Hayashi M., Lanagan M.T., Agrawal D.K., Nagata K. (2011). Complex Permittivity of Graphite, Carbon Black and Coal Powders in the Ranges of X-band Frequencies (8.2 to 12.4 GHz) and between 1 and 10 GHz. ISIJ Int..

[49] Wang F., Zhou Q., Zhang Z., He P., Zhang J., Jiang K. (2022). Microwave Absorption Performance of Carbon Black/Polylactic Acid Composite for Fused Filament Fabrication. Appl. Sci..

[50] Lambert C., Bumbaroska M., Dobson D., Hangas J., Pakko J., Tennison P. (2016). Analysis of High Mileage Gasoline Exhaust Particle Filters. SAE Int. J. Engines.

[51] Bromberg L., Sappok A., Koert P. (2013). Method and System for Controlling Filter Operation. US Patent application.

[52] Dietrich M., Hagen G., Reitmeier W., Burger K., Hien M., Grass P., Kubinski D., Visser J., Moos R. (2017). Radio-Frequency-Based NH_3_-Selective Catalytic Reduction Catalyst Control: Studies on Temperature Dependency and Humidity Influences. Sensors.

[53] Popovicheva O.B., Persiantseva N.M., Tishkova V., Shonija N.K., Zubareva N.A. (2008). Quantification of water uptake by soot particles. Environ. Res. Lett..

